# Can we include dichotomous variables in meta-analytic structural equation modeling? Mind the prevalence

**DOI:** 10.3758/s13428-025-02928-4

**Published:** 2026-03-30

**Authors:** Hannelies de Jonge, Belén Fernández-Castilla, Suzanne Jak, Kees-Jan Kan

**Affiliations:** 1 Methods and Statistics, Research Institute of Child Development and Education, University of Amsterdam, Amsterdam, The Netherlands; 2 Methodology and Statistics, Psychology, Leiden University, Wassenaarseweg 52, 2333 AK Leiden, The Netherlands; 3 Methodology of Behavioral Sciences, Psychology, Universidad Nacional de Educación a Distancia, Madrid, Spain

**Keywords:** Meta-analytic structural equation modeling, Cohen’s *d*, Point-biserial correlation, *d*-to-*r*_pb_ conversions, Dichotomous variables

## Abstract

Meta-analytic structural equation modeling (MASEM) is a method to systematically synthesize results from primary studies, allowing the researchers to simultaneously examine multiple relations among variables by fitting a structural equation model to the pooled correlations. Incorporating dichotomous variables (e.g., having a specific disease or not) into MASEM poses challenges. While primary studies that investigate the relation between a dichotomous and continuous variable typically report standardized mean differences (e.g., Cohen’s *d*), in the specialized MASEM software it is not possible to directly include standardized mean differences. Instead, MASEM typically uses correlation matrices as input. A proposed solution is to convert the standardized mean differences to point-biserial correlations. Here lies a complication because, in contrast to a standardized mean difference, the point-biserial correlation depends on the distribution of group membership. Through three Monte Carlo simulation studies, we investigated which conversion formula is suitable when one wants to include a dichotomous variable in MASEM. We varied the prevalence, sampling plan, within-study sample sizes, and the distribution of participants over two groups. Our results show that which conversion is suitable, and which is not depends on the aim of the meta-analyst. Moreover, if the group distribution in the sample does not reflect the prevalence in the population, it is necessary to adjust the correlation between the continuous variables in the model. We have extended our freely available web application (Effect Size Calculator and Converter; https://hdejonge.shinyapps.io/ESCACO/) to fill the existing gap and to assist the meta-analyst with both the conversions and the adjustment.

## Resumen en Español

El meta-análisis con modelos de ecuaciones estructurales (MASEM, por sus siglas en inglés, Meta-Analytic Structural Equation Modeling) es un método para sintetizar resultados de estudios primarios que permite examinar simultáneamente múltiples relaciones entre variables mediante el ajuste de modelos de ecuaciones estructurales sobre las correlaciones combinadas. Puesto que la unidad de análisis en MASEM son las correlaciones de Pearson, incorporar variables dicotómicas (e.g., tener o no una enfermedad) presenta desafíos. Los estudios primarios que analizan la relación entre una variable dicotómica y una continua suelen informar de diferencias de medias estandarizadas (e.g., *d* de Cohen), pero el software especializado de MASEM no permite incluirlas directamente. Una posible solución es convertir las diferencias de medias estandarizadas en correlaciones biseriales-puntuales. Sin embargo, esta transformación puede ser problemática, ya que, a diferencia de la diferencia de medias, correlación biserial-puntual depende de la distribución de los participantes en los grupos. El objetivo de este trabajo es investigar qué fórmula de conversión es adecuada para incluir una variable dicotómica en MASEM. Para ello, se realizaron tres estudios de simulación Monte Carlo en los que se variaron la prevalencia, el plan de muestreo, los tamaños muestrales y la distribución de participantes entre los grupos. Nuestros resultados muestran que la conversión más adecuada depende del objetivo del meta-analista. Además, si la distribución muestral no refleja la prevalencia poblacional, es necesario ajustar la correlación entre las variables continuas del modelo. Hemos ampliado nuestra aplicación web gratuita (Effect Size Calculator and Converter; https://hdejonge.shinyapps.io/ESCACO/) para cubrir esta necesidad y facilitar tanto las conversiones como el ajuste.

## Introduction

Researchers often want to compare whether two groups, for example, those with and those without a specific disease, differ on a quantitative variable (e.g., loneliness or distress). In such studies, researchers typically use mean differences to provide insight into how much (on average) individuals with the disease differ on the quantitative variable compared to those without the disease. Across primary studies, researchers may use different measurement instruments (perhaps with different scales) to measure the same quantitative variable. In order to ensure that the mean difference between the two groups on the quantitative variable is scale independent, it is typically expressed as a standardized mean difference (e.g., Cohen’s *d* or Hedges’ *g*). The standardized mean difference is the difference in means between the groups expressed in the number of standard deviations. An advantage of such standardization is that it is possible to compare different studies in terms of the magnitude of the effect, regardless of the instrument that is used to measure the quantitative variable.

**Fig. 1:**
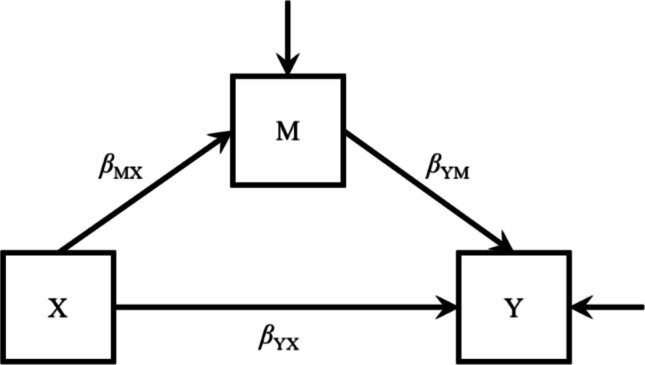
Path model representing partial mediation

With meta-analysis, it is possible to systematically synthesize the evidence from a collection of primary studies to determine the state of the art on a specific subject. Such a meta-analysis does not need to be limited to a univariate meta-analysis, where the focus is on the mean difference of a single variable. Of greater interest is the investigation of relationships between multiple variables, involving more complex hypotheses, such as in the mediation model in Fig. 1. Consider, for example, the following hypotheses. Individuals with breast cancer experience higher levels of loneliness than individuals without breast cancer, and because such feelings typically lead to greater emotional distress, individuals with breast cancer also experience, indirectly, more distress. To investigate such a mediation hypothesis, a possible technique to consider is correlation-based meta-analytic structural equation modeling (MASEM; Becker, 1992, 1995; Cheung & Chan, 2005; Jak et al., 2021; Viswesvaran & Ones, 1995). This technique would be the most suitable for the meta-analytic evaluation of indirect effects (Van Zundert & Miočević, 2020).

Generally, MASEM is a statistical method that combines meta-analysis with structural equation modeling. This method allows for the simultaneous investigation of multiple relationships within a single model by fitting a structural equation model to a meta-analytic dataset. One advantage of MASEM is the possibility of investigating new hypotheses that were not examined at the primary study level. Moreover, studies that investigated only some, but not all, of the relationships within the hypothesized model can be included in the meta-analysis.

During the meta-analytic data collection, the meta-analyst will commonly rely on reported summary data from primary studies rather than raw data, given the potential difficulty in obtaining the latter (Gabelica et al., 2022; Wicherts et al., 2006). As a result, meta-analysts often encounter a variety of effect sizes and need to convert one effect size or statistic to the other to arrive at a single type of effect size that can be summarized. Apart from the standardized mean difference discussed above, another popular effect size is the correlation coefficient. As noted above, when comparing groups, researchers typically report standardized mean differences, such as Cohen’s *d* and Hedges’ *g,* while MASEM is typically correlation-based. This poses a challenge for those who want to apply MASEM, because in the specialized MASEM software (i.e., R package metaSEM, Cheung, 2015), it is not possible to directly include standardized mean differences.

One proposed solution is to convert the standardized mean difference into a point-biserial correlation coefficient (*r*_pb_). This approach was the focus in prior research, where we aimed to investigate the incorporation of randomized controlled trial (RCT) data into MASEM (De Jonge et al., 2025). In this research, we showed that the relationship between a standardized mean difference and a point-biserial correlation coefficient can be expressed in multiple ways (see Table 1). Three of these expressions appear often in meta-analytic handbooks and mainstream conversion software. However, the third expression (i.e., Eq. 3, the so-called equal-groups conversion) is rare to find in the literature (but see Aaron et al. (1998) for the derivation). We note that we focused on RCTs, where the dichotomous variable reflected whether individuals were assigned to the experimental group or the control group. In such studies, expressions assuming equal group sizes yielded the most accurate point-estimates (while the standard errors were largely independent of conversion). This fits the assumption in RCTs that the population is split into two infinite, equally sized groups. The reason why these expressions outperform expressions without the assumption of equal groups is that when the groups in RCTs are not exactly equal in size, the formulas that include the assumption “adjust” the point-biserial correlation for unequal group sizes.

**Table 1:** Overview of *d-to-* ${r}_{\mathrm{pb}}$ conversion formulas

*d*-to-*r*_*pb*_ conversion	General (*t*-based)	In the limit (*z*-based)
Sample proportion(p)-based	${{r}_{\mathrm{pb}}}= \frac{d}{\sqrt{{d}^{2}+ \frac{{{(n}_{1} + {n}_{0})}^{2}-2({n}_{1} + {n}_{0})}{{n}_{1}{n}_{0}}}}$ (1)	${{r}_{\mathrm{pb}}}\approx \frac{d}{\sqrt{{d}^{2}+ \frac{{({n}_{1} + {n}_{0})}^{2}}{{n}_{1}{n}_{0}}}}$ (4)
Prevalence(π)-based	${{r}_{\mathrm{pb}}}=\frac{d}{\sqrt{{d}^{2}+ \frac{ 1 }{{\pi }\left(1- \pi \right) } - \frac{2 }{\pi \left(1- \pi \right)N}}}$ (2)	${r}_{\mathrm{pb}}\approx \frac{d}{\sqrt{{d}^{2}+ \frac{ 1 }{{\pi }\left(1- \pi \right)}}}$ (5)
Equally sized groups	${{r}_{\mathrm{pb}}}= \frac{d}{\sqrt{{d}^{2}+ 4-\frac{8}{N}}}$ (3)	${{r}_{\mathrm{pb}}}\approx \frac{d}{\sqrt{{d}^{2}+ 4}}$ (6)

*d* = Cohen’s *d*, ${r}_{\mathrm{pb}}$ = point-biserial correlation, ${n}_{0}$ = sample size of one group in a primary study, ${n}_{1}$ = sample size of the other group in the same primary study,* N* = ${n}_{0}$ + ${n}_{1}=$ total sample size in the primary study, π = prevalence expressed as a proportion

It is important to stress that the previous results applied only to RCTs or to situations where in the population two groups can be considered equally large (i.e., the population base rate is .50). We expect that the usage of the equal-groups conversion may result in biased estimates, when the group membership distribution in the population departs from a .50 probability and when the aim is to generalize findings towards the population. This is because, in contrast to a standardized mean difference, the point-biserial correlation depends on the group distribution. When referring to the population, this dependency (of *ρ*_pb_ on the group distribution) is known as population base rate^1^ sensitivity (McGrath & Meyer, 2006).

In settings other than in RCTs, the dichotomous variable is unlikely to be distributed evenly in the population. In other words, the population base rate for many dichotomous variables is rarely .50. One might consider scenarios in which dichotomous variables indicate whether an individual is employed or unemployed, is pregnant or not, has siblings or not, has a specific disease or not, and smokes or not. In these scenarios, the inference should not be towards the situation where groups are equal in size (hence a population base rate of .50). Rather, it should be towards the known or estimated population base rate (i.e., prevalence, denoted as π). Accordingly, when converting a standardized mean difference to a point-biserial correlation in a meta-analysis, it seems essential to use a conversion that accounts for the prevalence.

**Fig. 2:**
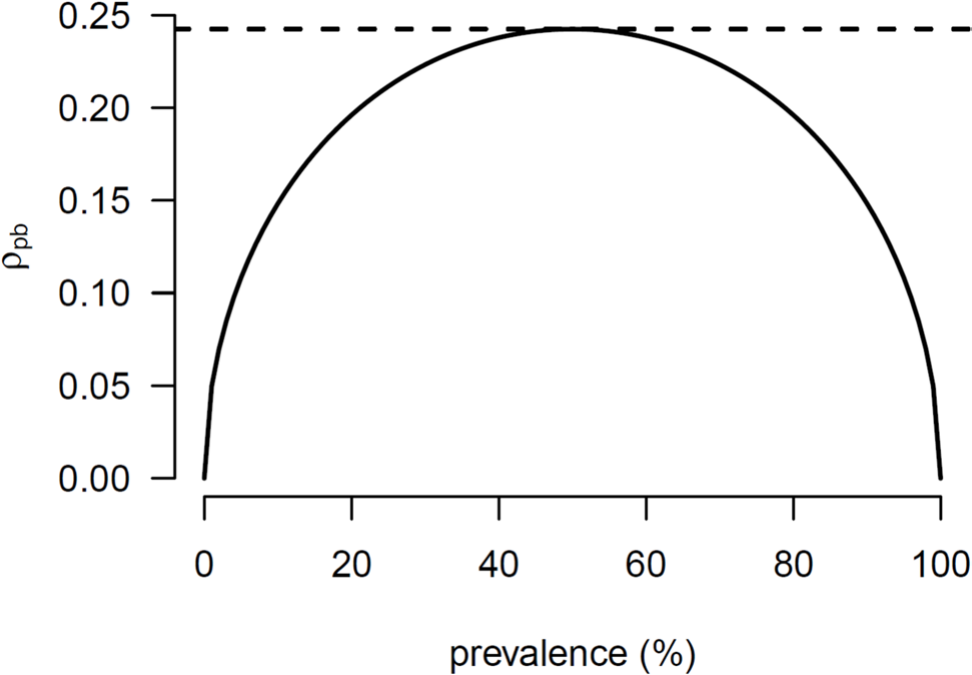
Dependence of ${\rho }_{\mathrm{pb}}$ on prevalence, assuming a standardized mean difference of 0.50

To illustrate the dependence of the ${\rho }_{\mathrm{pb}}$ (i.e., point-biserial correlation in the population) on the prevalence consider Fig. 2. Suppose the standardized mean difference in the population is 0.50, and the prevalence of breast cancer is 2% (i.e., π = .02). In this scenario, the ${\rho }_{\mathrm{pb}}$ equals .07. However, if the prevalence were 25%, the ${\rho }_{\mathrm{pb}}$ would be .21. Figure 2 also shows ${\rho }_{\mathrm{pb}}$ reaches its maximum absolute value when the prevalence is 50%.

Next to the dependence of the ${\rho }_{\mathrm{pb}}$ on the prevalence itself, the sampling procedure employed in the studies becomes relevant. Under simple random sampling, the sample proportion of X = 1 (e.g., having breast cancer) provides, in principle, an estimate of the population value, supporting the use of conversions based on the observed proportion. In other words, the occurrence of X = 1 (e.g., having breast cancer) in the sample will reflect the occurrence of X = 1 in the population (i.e., the prevalence of breast cancer). This suggests a conversion based on the proportion of X = 1 in the sample is applicable. With other sampling plans, such as, for example, 1:1 stratified sampling, the sample proportion does *not* provide an estimation of the distribution in the population. The point-biserial correlation based on the group sizes in the sample cannot be used to estimate the point-biserial correlation in the population. This suggests that the conversion based on the group sizes in the sample is not applicable to convert the standardized mean difference to a point-biserial correlation. The dependency of *r*_pb_, as an estimate of *ρ*_pb, _on the sampling procedure has implications for the meta-analyst who is interested in analyzing correlations rather than standardized mean differences. To illustrate these implications and the role of the different conversion formulas on the estimation of MASEM parameters under different prevalence scenarios and sample distributions, we conducted a series of Monte Carlo simulation studies. Before explaining these simulation studies in more detail, we first provide an overview of the various methods of converting the standardized mean difference to a point-biserial correlation.

The starting point is the conversion of the standardized mean difference ($\delta$) to the point-biserial correlation (${\rho }_{\mathrm{pb}}$) at the population level: ${\rho }_{\mathrm{pb}}=\frac{\delta }{\sqrt{{\delta }^{2}+ \frac{1}{\pi (1-\pi )}}}$ (Bonett, 2020). Substituting the sample standardized mean difference (Cohen’s *d*) results in several expressions of the sample point-biserial correlation (${r}_{\mathrm{pb}})$. 

## Sample proportion (p)-based general conversion

Method 1 is a conversion method we call sample proportion (p)-based general conversion (henceforth, p-based general conversion)*.* This method is described, for example, in Lakens (2013) and Jacobs and Viechtbauer (2017) and implemented in R package metafor (Viechtbauer, 2010):

1 $${{r}_{\mathrm{pb}}}= \frac{d}{\sqrt{{d}^{2}+ \frac{{{(n}_{1} + {n}_{0})}^{2}-2({n}_{1} + {n}_{0})}{{n}_{1}{n}_{0}}}}= \frac{d}{\sqrt{{d}^{2}+ \frac{ 1 }{{p}\left(1- p\right) } - \frac{2 }{p(1- p) N}}}.$$

Here, ${n}_{0}$ and ${n}_{1}$ refer to the group sizes in the sample while* N* is the total group size (*p* = sample proportion = $\frac{{n}_{1}}{N}$). Using this formula to convert the standardized mean difference (Cohen’s *d*; Eq. 7) to a point-biserial correlation, one would provide an ${r}_{\mathrm{pb}}$ for each primary study, which is dependent on that particular study’s sample group distribution.

## Prevalence (π)-based general conversion

If we substitute the sample proportion *p* with the prevalence, π (i.e., the population proportion), this results in Method 2:

2 $${r}_{\mathrm{pb}}=\frac{d}{\sqrt{{d}^{2}+ \frac{ 1 }{{\pi }\left(1- \pi \right) } - \frac{2 }{\pi (1- \pi ) N}}}.$$

The π in this method is either the known or assumed prevalence of the category coded as 1 in the dichotomous variable, for example, the prevalence of a specific disease. This expression makes the conversion independent of the group distribution in the samples of the primary studies (i.e., how the sample is divided into groups), and only depends on the known or assumed distribution in the population. We term this conversion the prevalence (π)-based general conversion (henceforth, π-based general conversion).

## Equal-groups conversion

If, for Method 1, we assume equal group sizes, or, equivalently, if we assume for Method 2 a prevalence of π = .50, we arrive at Method 3 (Aaron et al., 1998), termed the equal-groups conversion.

3 $${{r}_{\mathrm{pb}}}= \frac{d}{\sqrt{{d}^{2}+ 4-\frac{8}{N}}}.$$

We note that this method is generally advised when RCT data are the focus of the MASEM analysis (De Jonge et al., 2025). It fits with the logic underlying RCTs that the population consists of two infinite, equally sized groups.

## Large-sample variants of the conversions

Methods 4–6 constitute expressions inspired on the expression of the conversion of a standardized mean difference, δ, to a point-biserial correlation, *ρ*_pb_, on the population level (see above). One may also regard them as large-sample variants of the previously mentioned conversions: When sample sizes approach infinity, Methods 4–6 equal, respectively, Methods 1–3.

Method 4, reported in, for example, Borenstein et al. (2009), Borenstein & Hedges (2019), Lipsey & Wilson (2001)^2^, is

4 $${{r}_{\mathrm{pb}}}\approx \frac{d}{\sqrt{{d}^{2}+ \frac{{\left({n}_{1} + {n}_{0}\right)}^{2}}{{n}_{1}{n}_{0}}}}=\frac{d}{\sqrt{{d}^{2}+ \frac{ 1 }{{p}\left(1- p\right) } }}.$$

We call Equation 4 the sample proportion(p)-based large-sample conversion (henceforth, the p-based large-sample conversion).

Method 5 is the large sample version of Method 2:

5 $${r}_{\mathrm{pb}}\approx \frac{d}{\sqrt{{d}^{2}+ \frac{ 1 }{{\pi }\left(1- \pi \right) }}}.$$

We call this method the prevalence(π)-based large-sample conversion (henceforth, the π-based large-sample conversion).

Method 6, which is suggested by, for example, Borenstein et al. (2009), Borenstein and Hedges (2019), and Lipsey and Wilson (2001), is obtained by substituting* p* = .50 in Eq. 4:

6 $${{r}_{\mathrm{pb}}}_{ }\approx \frac{d}{\sqrt{{d}^{2}+ 4}}$$

We call this conversion the large-sample-equal-groups conversion.

## Present study

The aim of this study is to illustrate, by means of Monte Carlo simulations, the suitability and limitations of *d*-to-${r}_{\mathrm{pb}}$ conversions when meta-analysts want to include a dichotomous variable in MASEM. We focus specifically on the impact of the conversions on the estimation of the following MASEM parameters: pooled correlations, path coefficients, and indirect effect. In the simulations, we assumed that the meta-analyst (1) has at their disposal the standardized mean differences of M and Y (Cohen’s *d*: *d*_M_ and *d*_Y_) as well as the total correlation between M and Y (*r*_YM_), (2) converts the Cohen’s *d*s to *r*_pb_s, and (3) aims to analyze the matrix containing *r*_pb,MX_, *r*_pb,YX_, and *r*_YM_. In our simulation studies, we varied the prevalence, sampling plan, group sample sizes, and objective of the meta-analyst. In this paper, we only report the means of the pooled correlations. Readers interested in the mean path coefficients and indirect effect are referred to Supplementary Materials S1. We also provide the main overview of the relative percentage bias (RB) in all parameters of interest. Detailed information on the RB is available in the Supplementary Materials S2 and S3, which present the RB in the parameters of interest when mean estimates are compared to the population values (S2) and when compared to the maximum absolute values (i.e., when π = .50; S3).

## Method

To investigate the performance of the different conversion methods, we conducted three simulation studies, covering a total of 18 conditions. Each of these studies focuses on a specific practical application related to the sampling plan used in the primary studies. In Study 1, we considered meta-analyzing primary studies that employed simple random sampling, a strategy commonly used in epidemiological studies, for example. In Study 2, we considered studies that use a 1:1 stratified sampling plan, such that the two groups included are equal or at least relatively close in size to each other. In Study 3, we considered meta-analysis of a mix of studies that employ simple and 1:1 stratified sampling. We will first outline the assumptions and parameter values common to all three simulation studies. Next, we detail their differences.

## Constant conditions across the three simulation studies

To arrive at realistic conditions, we used empirical findings to design our simulation studies. In all three simulation studies and their conditions, we set the number of primary studies in the meta-analysis to 30. This number approximates the median number of primary studies in meta-analyses in behavioral and social sciences (39), biological sciences (32), and medical sciences (23) (Fernández-Castilla et al., 2020). Typically, in MASEM, not all primary studies in a meta-analysis investigate every variable or relationship in the hypothesized MASEM model (Sheng et al., 2016). On average, there are 39% missing correlations in studies that conduct MASEM. Therefore, in all our simulation studies and conditions, we aimed for a missingness as close as possible to 39%.

The structural equation model chosen to conduct our simulation studies was a standard mediation model containing three variables (see Fig. 1). Variable X was the dichotomous (grouping) variable and independent variable, variable M a continuous mediator, and Y a continuous outcome variable. The variables M and Y were multivariate normally distributed. We modeled X as having a direct effect on M; the effect of X on Y was predominantly indirect, although a direct path from X to Y was also specified in the model. To accomplish this, we chose a larger population standardized mean difference on M (${\updelta }_{\mathrm{M}}$= 0.80) than on variable Y (${\updelta }_{\mathrm{Y}}$ *=* 0.50). According to Cohen (1988) these values represent, respectively, a large and medium effect^3^. For the relationship between the variables M and Y, we assumed a within-group Pearson’s product–moment correlation of .40 in both groups (${\rho }_{\mathrm{YM},1}={\rho }_{\mathrm{YM},0}=.40=\rho$). We simulated data with standardized mean differences δ_M_ = 0.80 and δ_Y_ = 0.50. Means and standard deviations for M and Y were set accordingly (μ_M,1_ = 0.40, μ_M,0_ = – 0.40, σ_M,1_ = σ_M,0_ = 1; μ_Y,1_ = 0.25, μ_Y,0_ = – 0.25, σ_Y,1_ = σ_Y,0_ = 1). These parameters represent averages across subpopulations, implying a random-effects model with between-study variance (τ^2^) alongside sampling variance. The τ^2^ values (0.10 for δ_M_ and δ_Y_; 0.02 for ρ) were chosen based on empirical studies (Fernández-Castilla et al., 2020; Van Erp et al., 2017). Each primary study was assumed to sample participants from a unique subpopulation, consistent with common MASEM simulation practice.

In all three simulations, X was a dichotomous variable with values 1 (e.g., having a specific disease) and 0 (e.g., not having that disease). We let the prevalence of X = 1 vary: 2%, 10%, and 50% (i.e., π = .02, π = .10, and π = .50, respectively). This variation was chosen because, in practice, the prevalence can differ substantially, depending in part on the nature of X. In practice, X can refer to having (versus not having) a relatively rare condition, such as a specific type of cancer, or it can refer to a variable with nearly equally distributed values, such as having or not having the Y chromosome. This range of prevalences can also be found in substantive research (e.g., Yankaskas, 2006). At a prevalence of 50%, the point-biserial correlation would reach its maximum absolute value (under the assumption that the continuous variable is normally distributed).

As can be shown (see Appendix A), the population values of the correlations and path coefficients differ as a function of the prevalence. Appendix A also gives the calculation of the expected values for the pooled correlations (i.e., *r*_MX_, *r*_YX_, *r*_YM_), path coefficients (i.e., ${\widehat{\upbeta }}_{\mathrm{MX}}$, ${\widehat{\upbeta }}_{\mathrm{YX}}$, and ${\widehat{\upbeta }}_{\mathrm{YM}}$), and the indirect effect of X on Y, depending on the different prevalences in the conditions. We used these population values to compare them to the estimates resulting from the meta-analyses of the primary studies, as provided by the R package metaSEM (Cheung, 2015), implemented in R (version 4.2.2; R Core Team, 2022).

## Simulation studies

The main distinction between the three studies lies in the sampling plan of the primary studies. Below, we describe these differences in more detail, and in Table 2, we provide an overview of the differences between the studies and conditions.

**Table 2:** Summarized overview of the characteristics in which the studies and their conditions differ from each other

Study	Condition	Sampling plan	π	Target sample size	n1/n0 (group sample size distribution) in primary studies
1	1	Simple	.02	1000	~20/980
	2	Simple	.10	1000	~100/900
	3	Simple	.50	1000	~500/500
2	4	Stratified ~1:1	.02	26	~13/13
	5	Stratified ~1:1	.10	26	~13/13
	6	Stratified ~1:1	.50	26	~13/13
	7	Stratified ~1:1	.02	128	~64/64
	8	Stratified ~1:1	.10	128	~64/64
	9	Stratified ~1:1	.50	128	~64/64
	10	Stratified ~1:1	.02	26 to 600	Approximately equal
	11	Stratified ~1:1	.10	26 to 600	Approximately equal
	12	Stratified ~1:1	.50	26 to 600	Approximately equal
	13	Stratified unbalanced	.02	128	Sampled from uniform distribution from .20 to .60
	14	Stratified unbalanced	.10	128	Sampled from uniform distribution from .20 to .60
	15	Stratified unbalanced	.50	128	Sampled from uniform distribution from .20 to .60
3	16	Mixed sampling plans	.02	Mixed (1000 & 26 & 128)	~20/980 & ~13/13 & ~64/64
	17	Mixed sampling plans	.10	Mixed (1000 & 26 & 128)	~100/900 & ~13/13 & ~64/64
	18	Mixed sampling plans	.50	Mixed (1000 & 26 & 128)	~500/500 & ~13/13 & ~64/64

Simple = simple random sampling, Stratified ~1:1= stratified random sampling with approximately equal groups, Mixed sampling plans = 1/3 of the primary studies used simple random sampling with a target sample size of 1000, 1/3 of the primary studies used stratified random sampling with approximately equal groups with a target sample size of 26, 1/3 of the primary studies used stratified random sampling with approximately equal groups with a target sample size of 128.

## Study 1: Meta-analysis of studies using simple random sampling

Study 1 comprised the first three conditions, with the only factor that varied between conditions being the prevalence ($\pi$) (see Table 2). In addition, we assumed that all studies used a simple random sampling plan. This would be common in epidemiological studies. Epidemiological studies often use such sampling plans, because (1) *random sampling* should give a better estimate than samples obtained through nonrandom sampling, and (2) by *simple* random sampling, one receives a direct estimate of the prevalence. We note that such studies require relatively large samples.

In order to simplify the interpretation of the simulation results, the target sample size in all studies was chosen* N* = 1000. This held for every replication in the simulations. As our own interest lies in the performance of MASEM (using the R package metaSEM), we added a number of complications that could affect MASEMs performance. These concerned (a) dropout of participants (such that not all primary studies ended up having 1000 participants) and (b) missing variables (meaning that not all primary studies report information on all three variables). Both dropout and missingness were assumed to be the result of random processes. We chose a maximum dropout rate of 20%, and whether this dropout was anticipated or not. To accomplish this, we sampled the group sizes from a uniform distribution with lower and upper bounds of 800 and 1200.

## Study 2: Meta-analysis of studies using stratified sampling

In Study 2, we considered a scenario where the primary studies employed a sampling plan different from simple random sampling. We opted for a scenario in which the meta-analyst is interested in synthesizing results from clinical trial studies. Participants in clinical trial studies are not sampled from the general population using simple random sampling because this would likely result in a small number of the target group (e.g., individuals with breast cancer) compared to the control group. Instead, the target group is typically sampled from a pool of individuals known to have the condition of interest (e.g., breast cancer). The control group is comprised of individuals who are, on average, similar to the individuals in the target groups. Ideally, the sampling plan would involve stratified random sampling and/or matching. Often, researchers aim for approximately equal groups, hence aim for* p* = .50. In most conditions (4 to 12) we opted for a sampling process creating approximately equal groups. We note that in general, the samples in clinical studies are not as large as in the previous scenario. This scenario is thus also relevant with respect to the conversion formulas that assume large sample sizes.

Effectively, the factors manipulated in Study 2 were thus sample size and the prevalence. We first systematically varied the target sample size, from 26 in all 30 studies (conditions 4–6) to 128 in all studies (conditions 7 to 9). The within-study sample size of 26 was considered to denote a small sample size. The sample size of 128 was determined through power calculations and applies to scenarios where a *d* of .50 (defined as a ‘medium effect’ by Cohen, 1988) can be identified with a power of at least .80, assuming a group size ratio of 1:1. As in Study 1, we assumed a dropout rate of 20% and sampled group sizes from uniform distributions.

Next, in order to create more ecologically valid scenarios, we also included conditions (i.e., conditions 10–12) in which the group sample sizes varied over the 30 primary studies included in the meta-analysis (from 26 to 600). The sample size of 600 is based on the study by Fernández-Castilla et al. (2020), which found that in medical science the 3rd quartile of the number of primary studies in meta-analyses is 601. In all these conditions, we assumed the two groups to be approximately equal in size, with differences in group sizes attributable only to random dropout.

To enhance ecological validity even further, we also created conditions where the primary studies displayed more pronounced differences in sample size ratio between the groups (i.e., unbalanced groups), comprising conditions 13–15. For these conditions, the target total sample size of all 30 primary studies was 128 (again, we chose a maximum dropout rate of 20% and sampled from a uniform distribution). The sample proportion was sampled from a uniform distribution ranging from .20 to .60. All in all, Study 2 included a total of 12 conditions (see Table 2).

## Study 3: Meta-analysis of studies using simple random sampling and stratified sampling

In Study 3, the goal was to illustrate the implications if the meta-analyst ignores the nature of the sampling and includes both epidemiological and clinical studies, for example. In this scenario, we assumed that the meta-analyst includes effects obtained from different types of studies (i.e., studies using simple random sampling and studies using stratified sampling) and that effects are simply aggregated over studies. In these conditions, we assumed one-third of the primary studies used a simple random sampling plan with a target within-study sample size of 1000 (large) and two third used 1:1 stratified random sampling resulting in about equal group sizes. In half of the studies that used 1:1 stratified random sampling, the target sample size was assumed 26 (small) and in the other half 128 (medium). Again, to ensure the within-study sample size was not exactly equal across the 30 primary studies in the meta-analysis, we sampled the sample size from a uniform distribution and assumed a maximum dropout rate of 20%. In total, Study 3 covered three conditions (16, 17, and 18). As in Study 1, the only factor that varied across these conditions was $\pi$ (see Table 2).

## Data generation

In all three studies, we used R (version 4.2.2; R Core Team, 2022) to generate the data, specifically employing the mvrnorm() function from the MASS package (Venables & Ripley, 2002). Parameter values for the studies were sampled from a multivariate normal distribution with the following mean vector: [δ_M_ = 0.80, δ_Y_ = 0.50, ρ_YM_ = .40] and diagonal between-studies variance matrix [${\tau }_{\delta \mathrm{M}}^{2}=0.10, {\tau }_{\delta \mathrm{Y}}^{2}=0.10$, ${\tau }_{\rho \mathrm{YM}}^{2}=0.02$].

Using those parameter values, we determined the means of the two groups and drew sample data from a multivariate normal distribution for every primary study, separately for the two groups. For each study, we merged the data from the two groups and added a dummy variable to indicate group membership (variable X). Then, in 12 of the 30 datasets, we randomly removed one variable, resulting in 40% of the correlations being missing.

In each sample, we calculated the bivariate Pearson’s product–moment correlation between continuous variables M and Y. We also calculated the sample Cohen’s *d*s on both M and Y using the following formula (Borenstein et al., 2009; Borenstein & Hedges, 2019; Lakens, 2013; Lipsey & Wilson, 2001):

7 $$d\boldsymbol{ }= \frac{{\overline{y}}_{1}- {\overline{y}}_{0}}{{s}_{p}},$$

with

8 $${s}_{p}=\sqrt{\frac{{(n}_{1}-1){s}_{1}^{2}+{(n}_{0}-1){s}_{0}^{2}}{{n}_{1}+{n}_{0}-2}}.$$

In these equations, subscripts 1 and 0 refer to the target group and the control group. Symbols ${\overline{y}}_{1}$ and ${\overline{y}}_{0}$ denote the group sample means of Y, ${s}_{1}$ and ${s}_{0}$ the group sample standard deviations, and ${s}_{p}$ the pooled standard deviation.

We then converted Cohen’s *d* to point-biserial correlations using the six methods of conversion as reviewed in the Introduction. In this way, for each replication and study, we created six different sample correlation matrices, hence six different meta-analytic datasets. For each meta-analytic dataset, we fitted the partial mediation model depicted in Fig. 1, applying one-stage random effects MASEM (OSMASEM; Jak & Cheung, 2020). To this end, we used the function osmasem() within the R package metaSEM (Cheung, 2015)^4^. From the output, we obtained the path coefficients and model-implied correlations^5^.

In each condition, the number of replications was 2000. This number of replications is consistent with our previous simulation studies on MASEM (i.e., De Jonge et al., 2020; De Jonge et al., 2025), as well as with other simulation studies on MASEM using between 100 and 2000 replications (e.g., Bilici et al., 2025; Cai & Fan, 2020; Gnambs & Sengewald, 2023; Oort & Jak, 2016; Ke et al., 2018; Moeyaert et al., 2017). If the estimation procedure failed to converge, we reran the model. Based on the software’s (i.e., R package metaSEM) convergence status we checked if replications had converged. In addition, we checked if the parameter estimates (i.e., correlations and path coefficients) were plausible, for example, not exceeding 1. The R scripts necessary for reproducing the results can be accessed on the Open Science Framework platform (OSF; https://osf.io/mkv65/).

## Evaluation criteria

To evaluate the effects of the different conversions, we will present summary statistics of the following parameters: the averages of the model-implied correlations, path coefficients, and indirect effect in each condition. We also calculated the RB in the model-implied correlations, path coefficients, and indirect effect. We used the following formula to calculate the RB in the pooled correlations:

9 $$\frac{\overline{r} -\uprho }{\uprho }\cdot 100\text{ \%},$$

where $\overline{r}$ represents the mean of the estimated pooled correlation across replications, and $\uprho$ denotes the true value of the pooled correlation.

The following formula was used to calculate the RB in the path coefficients:

10 $$\frac{\overline{\widehat{\upbeta }} -\upbeta }{\upbeta }\cdot 100\text{ \%},$$

where $\overline{\widehat{\upbeta }}$ denotes the mean of the estimated path coefficients across replications, and $\upbeta$ the true value of the path coefficient. This true value depends on the prevalence assumed. Based on Hoogland and Boomsma (1998), we considered RB values below 5% as acceptable. This criterion is typically applied in simulation studies on meta-analysis (e.g., Cai & Fan, 2020; Gnambs & Sengewald, 2023; Moeyaert et al., 2017).

The RB in the indirect effect (i.e., ${\upbeta }_{\mathrm{MX} }\cdot {\upbeta }_{\mathrm{YM}}$) was estimated in the same way. Given that the indirect effect is the product of two direct effects, we considered RB values below 10.25% to be acceptable (because 1.05 ⋅ 1.05 = 1.1025).

## Results

In this section, we present the findings of each of the three simulation studies separately.

## Study 1: Meta-analysis of studies using simple random sampling

Table 3 contains the mean correlations observed across the 2000 replications in conditions 1 to 3, disaggregated by the prevalence (note the dependency of the population values of the point-biserial correlations on the prevalence). As can be observed, the equal-groups variant conversions (i.e., equal-groups and large-sample-equal-groups conversion) did not yield estimates of the population values (unless π = .50), while all other conversions did. The π-based conversions (i.e., π-based large-sample conversion and π-based general conversion) provided the closest estimates, but the p-based conversions (i.e., p-based large-sample conversion and p-based general conversion) also performed well (i.e., RBs within the 5% limit, see Supplementary Materials S2). As can be expected with sample sizes around 1000, the large sample variants (i.e., p-based large-sample conversion and π-based large-sample conversion) showed results near identical as compared to the general conversions (i.e., π-based and p-based). As one can notice and may expect, *r*_YM_ is not affected by the manner of conversion of Cohen’s *d*s.

**Table 3:** Means of the model-implied correlations across 2000 replications for Study 1, conditions 1 through 3 (simple random sampling)

	π =.02			π =.10			π =.50		
Population values	ρ_pb,MX_	ρ_pb,YX_	ρ_YM_	ρ_pb,MX_	ρ_pb,YX_	ρ_YM_	ρ_pb,MX_	ρ_pb,YX_	ρ_YM_
	.111	.070	.404	.233	.148	.419	.371	.243	.450
Estimations	*r*_pb,MX_	*r*_pb,YX_	*r*_YM_	*r*_pb,MX_	*r*_pb,YX_	*r*_YM_	*r*_pb,MX_	*r*_pb,YX_	*r*_YM_
p-based G	.111	.069	.404	.230	.145	.416	.361	.234	.440
π-based G	.111	.070	.404	.230	.145	.416	.361	.234	.440
EG	.358	.231	.404	.360	.233	.416	.361	.234	.440
p-based LS	.110	.069	.404	.230	.145	.416	.360	.234	.440
π-based LS	.111	.070	.404	.230	.145	.416	.360	.234	.440
LSEG	.358	.231	.404	.360	.232	.416	.360	.234	.440

*Note.* p-based G = p-based general conversion; π-based G = π-based general conversion; EG = equal-groups conversion; p-based LS = p-based large-sample conversion; π-based LS = π-based large-sample conversion; LSEG = large-sample-equal-groups conversion

In Study 1, we assumed an epidemiological objective and the aim to generalize results to the population. Taking that into account, the RB in the estimated correlations remained below 5% using p-based and π-based conversions. As a result of the model-implied correlations being unbiased, the RB in the path coefficients and indirect effect that were based on these correlations also remained below their limits (of 5% and 10.25%, respectively). We note that if the meta-analyst had used the equal-groups conversion variants, the bias in the point-biserial correlations would have become seriously large (e.g., more than 200%). As the path coefficients are functions of the correlations, the path coefficients would also have become seriously biased.

## Study 2: Meta-analysis of studies using stratified sampling

In Study 2, we used stratified random sampling, while prevalence (π), sample sizes, and sample group distribution varied. Tables 4, 5, 6, and 7 provide the mean correlations across the 2000 replications segregated by prevalence, where each table includes the results of a set of conditions based on sample group distribution and the sample size of the primary studies included in the meta-analysis. Across these tables we observe a common pattern. Independent of sample size and sample group distribution, when the prevalence is 50%, the equal-groups conversion and the π-based general conversion estimated correlations are generally closest to the values in the population. The RB remained below 5% using these two conversion methods. As a result, the RB in the path coefficients and indirect effect that were based on those correlations also remained below 5% and 10.25%, respectively. The π-based general conversion and equal-groups conversion gave the same results because the expressions of these two conversions are mathematically equal to each other when π = .50. The other conversions yielded slightly biased parameter estimates in a limited number of conditions (RBs just above 5%, see Supplementary Materials S2).

**Table 4:** Means of the model-implied correlations across 2000 replications for Study 2, conditions 4 to 6 (approximately 1:1 ratio and target within-study sample size = 26)

	π = .02			π = .10			π = .50		
Population values	ρ_pb,MX_	ρ_pb,YX_	ρ_YM_	ρ_pb,MX_	ρ_pb,YX_	ρ_YM_	ρ_pb,MX_	ρ_pb,YX_	ρ_YM_
	.111	.070	.404	.233	.148	.419	.371	.243	.450
Estimations	*r*_pb,MX_	*r*_pb,YX_	*r*_YM_	*r*_pb,MX_	*r*_pb,YX_	*r*_YM_	*r*_pb,MX_	*r*_pb,YX_	*r*_YM_
p-based G	.361	.234	.434	.358	.231	.433	.359	.231	.430
π-based G	.119	.074	.434	.240	.152	.433	.364	.234	.430
EG	.366	.237	.434	.364	.235	.433	.364	.234	.430
p-based LS	.350	.226	.434	.348	.224	.433	.348	.223	.430
π-based LS	.114	.072	.434	.231	.146	.433	.353	.227	.430
LSEG	.356	.230	.434	.353	.227	.433	.353	.227	.430

p-based G = p-based general conversion; π-based G = π-based general conversion; EG = equal-groups conversion; p-based LS = p-based large-sample conversion; π-based LS = π-based large-sample conversion; LSEG = large-sample-equal-groups conversion

**Table 5:** Means of the model-implied correlations across 2000 replications for Study 2, conditions 7 to 9 (approximately 1:1 ratio and target within-study sample size = 128)

	π = .02			π = .10			π = .50		
Population values	ρ_pb,MX_	ρ_pb,YX_	ρ_YM_	ρ_pb,MX_	ρ_pb,YX_	ρ_YM_	ρ_pb,MX_	ρ_pb,YX_	ρ_YM_
	.111	.070	.404	.233	.148	.419	.371	.243	.450
Estimations	*r*_pb,MX_	*r*_pb,YX_	*r*_YM_	*r*_pb,MX_	*r*_pb,YX_	*r*_YM_	*r*_pb,MX_	*r*_pb,YX_	*r*_YM_
p-based G	.361	.235	.439	.361	.234	.438	.361	.235	.439
π-based G	.112	.071	.439	.233	.148	.438	.362	.236	.439
EG	.362	.236	.439	.362	.235	.438	.362	.236	.439
p-based LS	.359	.233	.439	.359	.233	.438	.359	.234	.439
π-based LS	.112	.070	.439	.231	.146	.438	.360	.235	.439
LSEG	.360	.234	.439	.360	.234	.438	.360	.235	.439

p-based G = p-based general conversion; π-based G = π-based general conversion; EG = equal-groups conversion; p-based LS = p-based large-sample conversion; π-based LS = π-based large-sample conversion; LSEG = large-sample-equal-groups conversion

**Table 6:** Means of the model-implied correlations across 2000 replications for Study 2, conditions 10 to 12 (approximately 1:1 ratio and target within-study sample sizes range between 26 and 600)

	π = .02			π = .10			π = .50		
Population values	ρ_pb,MX_	ρ_pb,YX_	ρ_YM_	ρ_pb,MX_	ρ_pb,YX_	ρ_YM_	ρ_pb,MX_	ρ_pb,YX_	ρ_YM_
	.111	.070	.404	.233	.148	.419	.371	.243	.450
Estimations	*r*_pb,MX_	*r*_pb,YX_	*r*_YM_	*r*_pb,MX_	*r*_pb,YX_	*r*_YM_	*r*_pb,MX_	*r*_pb,YX_	*r*_YM_
p-based G	.361	.235	.438	.361	.235	.439	.362	.235	.438
π-based G	.111	.070	.438	.231	.147	.439	.363	.235	.438
EG	.361	.236	.438	.361	.235	.439	.363	.235	.438
p-based LS	.359	.234	.438	.359	.234	.439	.361	.234	.438
π-based LS	.111	.070	.438	.230	.147	.439	.361	.234	.438
LSEG	.360	.235	.438	.360	.234	.439	.361	.234	.438

p-based G = p-based general conversion; π-based G = π-based general conversion; EG = equal-groups conversion; p-based LS = p-based large-sample conversion; π-based LS = π-based large-sample conversion; LSEG = large-sample-equal-groups conversion

**Table 7:** Means of the model-implied correlations across 2000 replications for Study 2, conditions 13 to 15 (Sample group distribution different for every study, but target within-study sample sizes always 128)

	π = .02			π = .10			π = .50		
Population values	ρ_pb,MX_	ρ_pb,YX_	ρ_YM_	ρ_pb,MX_	ρ_pb,YX_	ρ_YM_	ρ_pb,MX_	ρ_pb,YX_	ρ_YM_
	.111	.070	.404	.233	.148	.419	.371	.243	.450
Estimations	*r*_pb,MX_	*r*_pb,YX_	*r*_YM_	*r*_pb,MX_	*r*_pb,YX_	*r*_YM_	*r*_pb,MX_	*r*_pb,YX_	*r*_YM_
p-based G	.346	.225	.435	.346	.224	.435	.345	.224	.434
π-based G	.112	.071	.435	.233	.148	.435	.361	.235	.434
EG	.362	.237	.435	.362	.236	.435	.361	.235	.434
p-based LS	.344	.224	.435	.344	.223	.435	.343	.222	.434
π-based LS	.112	.070	.435	.231	.147	.435	.358	.234	.434
LSEG	.360	.235	.435	.360	.234	.435	.358	.234	.434

p-based G = p-based general conversion; π-based G = π-based general conversion; EG = equal-groups conversion; p-based LS = p-based large-sample conversion; π-based LS = π-based large-sample conversion; LSEG = large-sample-equal-groups conversion

When the prevalence deviated from 50%, the conversions yielded results that were more clearly disparate from each other. The π-based conversions yielded estimates of the actual *ρ*_pb,MX_ and *ρ*_pb,YX_, while the other variants provided estimates of the maximum absolute point-biserial correlations. When using the π-based conversions, the RB values of *r*_pb,MX_ and *r*_pb,YX_ typically remained below 5%. However, the means of ${\widehat{\upbeta }}_{\mathrm{YX}}$ and ${\widehat{\upbeta }}_{\mathrm{YM}}$, which are functions of the correlations, were typically more than 5% biased, up to (–)15.33% (see supplementary materials S2). The RBs in ${\widehat{\upbeta }}_{\mathrm{MX}}$ and the indirect effect remained often below 5% and 10.25%, respectively. We note that conversions other than the π-based conversions showed substantial bias in the model-implied correlations, path coefficients, and indirect effect (e.g., RBs above 200%).

From the results in Tables 4, 5, 6, and 7, one may notice that the estimation of Pearson’s product–moment correlation between the two continuous variables M and Y (${\rho }_{\mathrm{YM}}$) did not depend on conversion methods. However, note that ${\rho }_{\mathrm{YM}}$ depends on π and that the mean values *r*_YM_ did not concern estimates of this population correlation (${\rho }_{\mathrm{YM}}$). Rather, they depend on the sample proportions. As in this simulation the group sizes were typically approximately equal to each other (p = .50), they concern estimates of a Pearson’s product–moment correlation pertaining to ${\rho }_{\mathrm{YM}}$ if the prevalence were 50% (π = .50). The maximum RB in *r*_YM_ across all conditions and all conversion was 8.479% (see Supplementary Materials S2). Taken together, when the sample proportion differs from the prevalence, the meta-analyst’s gathered information may result in biased *r*_YM_ when extrapolating to the population. This, in turn, affects the estimation of the path coefficients in the model. We explain this issue, as well as a solution, more elaborately in the discussion section.

Rather than estimating the actual population parameter values, one may wish to estimate the maximum absolute values of the point-biserial correlations. These can be obtained by the equal-groups conversion. Compared to the true maximum absolute values, the results showed that the use of the equal-groups conversion yields estimates that can be considered unbiased, not depending on whether the group sizes are balanced or unbalanced (i.e., the RBs remain below the 5% and 10.25% criterion; see Supplementary Materials S3).

## Study 3: Meta-analysis of studies using simple random sampling and stratified sampling

In Study 3, we considered the scenario where the meta-analyst does not take into account the different sampling plans and includes all studies, irrespective of whether these used simple or stratified sampling plans. In Table 8, we report the mean correlations observed across the 2000 replications disaggregated by the population prevalence. As can be observed, the equal-groups variant conversions (i.e., equal-groups and large-sample-equal-groups conversion) yielded again estimates of the maximum absolute values of the point-biserial relations (i.e., *r*_pb,MX_ and *r*_pb,YX_), while the π-based conversions (i.e., π-based general conversion and π-based large-sample conversion) yielded estimates of the population values. The RB values were consistently below 5% (see supplementary materials S2 for RB values when the reference value was the population value and S3 for RB values when the reference value was the maximum absolute value).

**Table 8:** Means of the model-implied correlations across 2000 replications for Study 3, conditions 16 to 18 (Mixed sampling plans and mixed target within-study sample sizes)

	π = .02			π = .10			π = .50		
Population values	ρ_pb,MX_	ρ_pb,YX_	ρ_YM_	ρ_pb,MX_	ρ_pb,YX_	ρ_YM_	ρ_pb,MX_	ρ_pb,YX_	ρ_YM_
	.111	.070	.404	.233	.148	.419	.371	.243	.450
Estimations	*r*_pb,MX_	*r*_pb,YX_	*r*_YM_	*r*_pb,MX_	*r*_pb,YX_	*r*_YM_	*r*_pb,MX_	*r*_pb,YX_	*r*_YM_
p-based G	.356	.181	.421	.358	.205	.426	.361	.235	.437
π-based G	.113	.070	.424	.234	.148	.428	.363	.236	.437
EG	.363	.234	.424	.363	.235	.428	.363	.236	.437
p-based LS	.351	.179	.421	.353	.203	.426	.356	.233	.437
π-based LS	.112	.070	.424	.231	.147	.428	.358	.234	.437
LSEG	.358	.232	.424	.358	.233	.428	.358	.234	.437

p-based G = p-based general conversion; π-based G = π-based general conversion; EG = equal-groups conversion. p-based LS = p-based large-sample conversion; π-based LS = π-based large-sample conversion; LSEG = large-sample-equal-groups conversion

Table 8 reveals that the discrepancy between the means of *r*_YM_ and the true population value (ρ_YM_) increased as the prevalence deviated more from the sample. The π-based conversions yielded unbiased estimates of the population values of *r*_pb,MX_ and *r*_pb,YX_. Estimates of the population values of *r*_YM_ were unbiased, irrespective of the conversion applied (see Supplementary Material S2). When the prevalence was 50%, the path coefficients and indirect effect were unbiased (no matter which conversion was used). However, when the prevalence deviated from 50%, bias was present in ${\widehat{\upbeta }}_{\mathrm{YX}}$ when applying the π-based conversions (up to approximately (–)10%; see Supplementary Materials S2). Other conversions yielded substantially stronger bias (e.g., more than 200%).

As noted above, when estimating the maximum absolute value of the point-biserial correlation instead of the population values, the equal-groups variants yielded unbiased *r*_pb,MX_ and *r*_pb,YX_ estimates. In addition, the conversions did not affect the estimation of the maximum absolute value of *r*_YM_ (RB values for *r*_YM_ were approximately equal across conversions; see Supplementary Material S3). When the prevalence was 50%, *r*_YM_ estimates were unbiased. However, as the prevalence decreased, *r*_YM_ estimates were not always unbiased anymore. The equal-groups variants yielded biased estimates of the maximum absolute values of *r*_YM_ when the prevalence became 2%. With respect to the path coefficient(s), bias was not only present when the prevalence was 2% but also when the prevalence was 10%. Applying the other conversion yielded substantial stronger bias in the path coefficient(s).

In conclusion, aggregating studies that use various sampling plans implies varying sample proportions across primary studies. In some studies, the sample proportions reflect the prevalence (when simple random sampling is used), while in others they are not and can be close to .50 (for example, when 1:1 stratified sampling is used). As a result of this variety in sampling proportions, the aggregated Pearson’s product–moment correlation between M and Y lay between the population value (*ρ*_YM_) and its absolute maximum (i.e., when π would have been .50). We present a solution to this problem in the next section.

## Discussion

To investigate the impact of various *d*-to-*r*_pb_ conversions on parameter estimations in MASEM, we conducted a series of Monte Carlo simulation studies. The results of these simulations indicate that the most appropriate conversion depends on the meta-analyst’s goal. After selecting a conversion method aligned with the meta-analyst’s objective, that approach should be applied consistently across all studies in a single meta-analysis. Using multiple conversion approaches may yield effect sizes that are not directly comparable and should therefore not be jointly synthesized in a meta-analysis due to interpretational inconsistencies.

## Meta-analysis of studies using simple random sampling

If the goal is to synthesize studies using simple random sampling, for example, in epidemiological studies, and the further aim is to generalize findings to the population, we recommend the π-based general conversion formula (Eq. 2), where π stands for the prevalence. Since the point-biserial correlation depends on prevalence, we recommend that meta-analysts report which value for π they used. If there is no prior knowledge about π or one is uncertain about it, one may use the p-based general conversion (Eq. 1), because in this case this also leads to unbiased parameter estimates. However, using the p-based general conversion might require some caution. In our simulation study, the number of primary studies (30) and the target sample size (1000) can be considered, respectively, medium and large. In this scenario, the outcomes of using the p-based general conversion were close to outcomes when using π-based conversions. This is in line with the Introduction, where we stated that when simple random sampling is successfully employed, the sample group distribution represents an approximation of the population group distribution (π). When the number of primary studies or sample sizes is smaller, the estimates might be less close to the population values. Further research is required to ascertain whether the p-based general conversion still leads to unbiased estimation of the population values when the number of primary studies and sample size are smaller than those investigated in our simulation.

When the goal is to synthesize studies using simple random sampling, and the further aim is to estimate the population parameters, we discourage the equal-groups variants of the conversion (Eq. 3 and Eq. 6), unless the prevalence is 50%. If the prevalence deviates from 50%, the use of the equal-groups variants can lead to seriously biased population parameter estimates. The larger the deviation from 50%, the larger the bias.

In our simulation, we assumed that each primary study sample was drawn from the same population. The sample in each study is then roughly split according to the population prevalence. In practice, it might be possible that primary studies sample from “subpopulations” with their own specific prevalence. For example, suppose the meta-analyst is interested in the effect of having breast cancer on the level of anxious feelings while the target population is Europe. Primary studies might include breast cancer patients from Spain, Denmark, Italy, or France (and so on). These are all subpopulations of individuals with breast cancer in specific European countries, while the prevalence of breast cancer may vary across these countries. To investigate whether our previously mentioned conclusions also hold in such a case, we conducted additional simulations (all R scripts needed to reproduce these additional simulations can be found on OSF; https://osf.io/mkv65/). In these extra simulations, we sampled a study-level (subpopulation) prevalence from a uniform distribution with a 30% range around the population π. For example, if the population π is .02, we sampled the prevalence from the interval [.014, .026]. This results in heterogeneity among the studies (also with respect to sample group distributions). The rest of the simulation was carried out in the same way as in Study 1 (simple random sampling), thus yielding three additional conditions (for values of π of .02, .10, .50). Again, we applied the six conversions as in our main study. However, we now use the π-based conversion formulas in two ways: once by plugging in the population π (as in the main study), and once by plugging in the subpopulation π (which varied across primary studies). This resulted in eight conversion methods, rather than six in the main study.

The results (i.e., means of and RB in all parameters of interest) for the three additional conditions are tabulated in Supplementary Materials S4 and S5. Here we provide the overall picture. Again, the π-based general conversion, using the population π, generally performs best. Using the subpopulation prevalences performed slightly less but still resulted in unbiased model-implied correlations, path coefficients, and indirect effect (i.e., RB values below 5%). The results actually typically lie between the results of the π-based conversion (using the population prevalence) and the sample proportion (p)-based conversion. Based on the extra simulations, we find stronger support for recommending the π-based general conversion.

The π-based general conversion requires knowledge of prevalence π, however. In cases where the population prevalence is unknown, but the meta-analyst has knowledge about the subpopulation prevalence for each primary study, these additional simulations suggest that the subpopulation values can be safely used instead. However, we want to emphasize the importance of meta-analysts clearly stating the prevalence they aim to generalize to. This improves transparency about the prevalence underlying the pooled correlations (and, consequently, the path coefficients and indirect effects), while also enhancing clarity and enabling easier comparison across different meta-analyses. If the population prevalence is unknown, one could consider meta-analyzing the subpopulation prevalences from the primary studies, or, at a minimum, report the subpopulation prevalences for each primary study included in the meta-analysis.

## Meta-analysis of studies using stratified sampling

If the goal is to synthesize studies using sampling plans other than simple random sampling, caution is required. In clinical trials, for example, 1:1 stratified sampling is common. In this scenario, the sample proportion (p) will approximate .50 and does typically *not* reflect the prevalence (π). Conversions that incorporate the prevalence (i.e., π-based conversions) can be viewed as adjustments that “correct” the point-biserial correlation between the dichotomous and continuous variable for inference to the population. The results of our study showed that, indeed, using the π-based general conversion typically leads to unbiased estimates of the point-biserial correlations between variable X and M and between X and Y. However, the π-based general conversion (like all other conversions) will not affect, hence not “adjust”, the relations between continuous variables, which may also figure in the hypothesized model (like in Fig. 1). Since the total correlation between M and Y, ${\rho }_{\mathrm{YM}}$, depends on prevalence, $\pi$ (see Appendix A), such a relation needs adjustment. Hence, the results of our study indicate that if the sample proportion differs from the prevalence, the estimates of path coefficients and indirect effects can become biased if such an adjustment is not applied.

Rather than estimating the actual population parameter values, the meta-analyst may be interested in estimating the maximum absolute value of the point-biserial correlations. This is possible with the equal-groups conversion (which is equal to substituting π = .50 in the π-based general conversion). The results of our simulation study indicate that even when all primary studies have unbalanced sample sizes, with the sample group distribution randomly sampled from a uniform distribution ranging from .20 to .60, this still leads to unbiased estimates for the relation between the two continuous variables. An “adjustment” of the relation between the continuous variables that are included in the hypothesized model does not seem to be required. However, it is important to note that when group sizes are more unbalanced, the sample proportion will deviate further from π = .50. This may lead to biased estimates for the relationship between the continuous variables, hence in principle “adjustment” of this relation is required.

As mentioned, the aim of the meta-analyst may be to estimate the maximum absolute point-biserial correlation. This maximum absolute point-biserial correlation can be interpreted as the effect size if both groups in the population were of equal size (instead of using the true π, π is imagined to be .50). One might be interested in this approach. Firstly, it is possible to compare the maximum absolute point-biserial correlations across meta-analyses examining different prevalences. Regardless of the actual prevalence, and given the same standardized mean difference, the maximum absolute value of the point-biserial correlation is always the same (under the assumption that the continuous variable is normally distributed). Secondly, the maximum absolute point-biserial correlation is an effect size that is, in principle, comparable to effect sizes from RCTs. Whether the effect size is comparable to RCTs in practice is a different question and relates not so much to whether simple or stratified sampling procedures have been employed, but to whether the sampling was random or nonrandom or to whether assignment to groups was random or nonrandom. Nonrandom sampling threatens external validity; nonrandom assignment provides threats to internal validity, such as selection effects and confounding. In RCTs, the randomized assignment protects against such complicating factors, but this protection is absent in the non-randomized designs we considered here. Because of such differences, a comparison between effects in randomized and non-randomized designs should only happen with caution. This is, of course, a general warning and has little to do with the application of MASEM.

## Meta-analysis of a mix of studies with different sampling plans

Our simulations indicate that combining studies with different sampling plans in a single MASEM analysis requires caution. The use of the equal-groups variant conversions will lead to accurate estimates of the maximum absolute values of the relationship between the dichotomous and continuous variable (i.e., point-biserial correlation). Additionally, the π-based conversions will lead to accurate estimates of the population values of the relationship between the dichotomous and continuous variable (i.e., point-biserial correlation, *r*_pb,MX_ and *r*_pb,YX_ in our study). However, “adjustment” is necessary for the relationship between the continuous variables (i.e., Pearson’s product–moment correlation, *r*_YM_ in our study). Without adjustment, the pooled Pearson product–moment correlation does not provide a clear estimate of either the population correlation or the maximum absolute correlation, which complicates interpretation and may introduce bias in the pooled Pearson product–moment correlation, path coefficients, and indirect effect. We discuss this adjustment in the next section. However, also with such adjustments, we caution against the aggregation of studies with different sampling plans. Different sampling plans could lead to different participant characteristics, which can compromise interpretability. Careful consideration of study differences remains essential. As an alternative, one may either include the sampling plan as a moderator in a single MASEM, which can adjust estimates for systematic differences between designs, or conduct separate analyses for each type of sampling plan, which allows correlations to be interpreted within each sampling design.

## Adjustment of the relation between the continuous variables in the mediation model

As described above, our results indicate that when the sample proportion differs from the population prevalence, the pooled Pearson product-moment correlation between the continuous variables may become biased. Because the path coefficients and indirect effect are functions of this correlation, such bias may propagate to the estimated path coefficients and indirect effect. Consequently, to obtain unbiased estimates of these parameters, an adjustment for the Pearson product–moment correlation between continuous variables is necessary. In the model in our simulations, the meta-analyst who has available the total Pearson product–moment correlation *r*_YM_, the standardized mean differences of M (*d*_M_) and Y (*d*_Y_), and the sample proportion, *p*, under which these values were obtained, could first calculate the within-group Pearson product–moment correlation (${r}_{\mathrm{W}}$), assuming that the within-group correlations are equal across the groups, using the following formula:

$${r}_{\mathrm{W}}= {r}_{\mathrm{YM}}\sqrt{1+ {d}_{\mathrm{M}}^{2} p\left(1-p\right)} \sqrt{1+ {d}_{\mathrm{Y}}^{2} p\left(1-p\right) }- {d}_{\mathrm{M}}{d}_{\mathrm{Y}} p\left(1-p\right) .$$

Next, this within-group correlation, Cohen’s *d*s, and π (i.e., the prevalence to which one aims to generalize) can be substituted in the following expression (see Appendix A).

$${\rho }_{\mathrm{YM}}=\frac{\pi \left(\rho +\left({\mu }_{\mathrm{M},1}-{\mu }_{\mathrm{M},\mathrm{T}}\right)\left({\mu }_{\mathrm{Y},1}-{\mu }_{\mathrm{Y},\mathrm{T}}\right)\right)+\left(1-\pi \right)\left(\rho +\left({\mu }_{\mathrm{M},0}-{\mu }_{\mathrm{M},\mathrm{T}}\right)\left({\mu }_{\mathrm{Y},0}-{\mu }_{\mathrm{Y},\mathrm{T}}\right)\right)}{{\sigma }_{\mathrm{Y},\mathrm{T}}{\sigma }_{\mathrm{M},\mathrm{T}}},$$

where $\rho$ stands for the within-group correlation, μ for the mean, and σ for the standard deviation (of variables M or Y, in groups 1 or 0 or combined, T), all at the population level. The adjusted total correlation, ${r}_{YM}^{*}$, then becomes:

$${r}_{YM}^{*}=\frac{\pi \left({r}_{\mathrm{W}}+\left({d}_{\mathrm{M}}-{\pi d}_{\mathrm{M}}\right)\left({d}_{\mathrm{Y}}-{\pi d}_{\mathrm{Y}}\right)\right)+\left(1-\pi \right)\left({r}_{\mathrm{W}}+\left(0-{\pi d}_{\mathrm{M}}\right)\left(0-{\pi d}_{\mathrm{Y}}\right)\right)}{\sqrt{1+{d}_{\mathrm{M}}^{2}\pi (1-\pi )}\sqrt{1+{d}_{\mathrm{Y}}^{2}\pi (1-\pi )}}$$

Hence, if we have a primary study with a 1:1 stratified sampling plan with exactly equal group sizes (*p* = .50), and the reported values of *d*_M_ = 0.80, *d*_Y_ = 0.50, and *r*_YM_ = .450 (which is population value when $\pi$ = .50), the adjusted correlation becomes ${r}_{\mathrm{YM}}^{*}$= .404 when plugging in $\pi$ = .02. This adjusted correlation is indeed the population value when $\pi$ = .02 (see Appendix A). To further investigate this adjustment formula, we applied the adjustment formula across all conditions in our main study as well as the three additional conditions described above (resulting in a total of 21 conditions), and reran the simulations. All R scripts needed to reproduce these simulations can be found on OSF; https://osf.io/mkv65/).

Under the assumption that the goal is to generalize toward the population, we focused only on the π-based conversion formulas (Eqs. 2 and 5), where we substitute either the population prevalence or the subpopulation prevalences, as the adjustment for the relationship between the continuous variables is only applied in these scenarios. The results (i.e., means of and RB in all parameters of interest) of these additional simulations can be found in Supplementary Materials S6 and S7. Focusing on the π-based general conversion with the population π substituted, which is the formula we previously recommended based on earlier results, we found that the model-implied correlations, path coefficients, and indirect effect were typically unbiased (i.e., RB values below 5%). One exception is when sample sizes are small (i.e., 26) while the prevalence is low (i.e., .02 and .10). In such circumstances, the bias can exceed the limit of 5% (up to 8% in our simulations).

For those interested in estimating the maximum value (i.e., when the prevalence to which one aims is .50), we focused on the equal-groups conversion formulas (Eqs. 3 and 6), as the adjustment for the relationship between the continuous variables was only applied there. The results (i.e., means of and RB in all parameters of interest) of these additional simulations can be found in Supplementary Materials S6 and S8. Regarding the equal-groups conversion (Eq. 3), which was our previously recommended formula, we found that the model-implied correlations, path coefficients, and indirect effect were typically unbiased. However, there are two exceptions: when simple random sampling is used with a population π of .02 in every primary study, or when the subpopulation π values differ per primary study and are sampled from a population π of .02. In these cases, we observed slightly biased estimates of β_YX_, with a maximum bias of – 5.49%.

Hence, the adjustment formula appears to work well. We therefore recommend that, when the sample proportion differs from the population prevalence to which one wants to generalize, this adjustment for the relationship between the continuous variables should be applied, at least for the specific model investigated in this study (i.e., a mediation model with three variables). Although we recommend using this adjustment formula, we want to note that applying it does not guarantee unbiased path coefficients under conditions more extreme than those examined in our simulation study. A complicating factor in practice when aiming to apply the aforementioned adjustment formula is that the required information may not be available for the meta-analyst. This is because in primary studies, they may not examine all variables or relationships in the hypothesized MASEM model (Sheng et al., 2016). In those situations, we caution against including dichotomous variables in MASEM when the sample proportion differs from the prevalence, and one aims to estimate population values. Another consideration is that applying the adjustment formula in other (e.g., more complex) models does not necessarily guarantee unbiased estimation of MASEM parameters. This highlights the need for further research into whether such adjustments – or other solutions – can be extended to other types of models that include dichotomous variables. Future simulation studies could investigate how these approaches affect the accuracy of MASEM parameter estimates in more extreme conditions and in models beyond the one examined in our study.

## Effect Size Calculator and Converter (ESCACO)

As described above, we recommend different conversions depending on the researcher’s aim. Some of these conversions have not been incorporated in conversion tools (for an overview of which formulas are implemented in which conversion tool, please see De Jonge et al., 2025). In addition, we have implemented the adjustment formula for Pearson’s product–moment correlation between continuous variables in the mediation model, as described above. To fill the existing gap and to assist the meta-analyst with their conversions and the adjustment formula, we have further developed our freely available web application, Effect Size Calculator and Converter (ESCACO). In general, ESCACO provides tools to support meta-analysts when including dichotomous (based on De Jonge et al., 2025, and this study) and dichotomized variables (based on De Jonge et al., 2020) in their hypothesized model. ESCACO is freely accessible online in the form of a Shiny app (https://hdejonge.shinyapps.io/ESCACO/) and offline as an R script on OSF (https://osf.io/mkv65/).

## General limitations and suggestions for further research

A general limitation of this study is that we have only investigated the impact of the *d*-to-*r*_pb_ on the MASEM parameters under the assumption that confounding and selection effects were absent and there were no other complicating factors, such as differences in variance between the groups. This helped us to understand and illustrate the role of the sampling plan employed, which was the focus of this study. In more realistic situations, it might be possible that the target group (e.g., individuals with a specific disease) differs from the control group. For example, individuals with a specific disease may exhibit greater similarity to one another, which could result in a reduction in variance.

What may be regarded as a possible limitation is that the number of primary studies included in the meta-analysis was always 30. In general, including a different number of primary studies could affect the estimation of the MASEM parameters estimates (s﻿pecifically on the accuracy of the standard errors; see De Jonge et al. (2025) and Jak and Cheung (2020)). However, the objective of this paper was not to investigate the specific strength of the bias or the number of primary studies required to ensure unbiased estimates. Further research is required to investigate this. Additionally, as noted above, the adjustment of the correlation between the continuous variables should be the subject of further research.

## Conclusion

The inclusion of a dichotomous variable in MASEM is possible but requires *d*-to-*r*_pb_ conversion. What conversion is suitable and what conversion is not depends on the meta-analyst’s goal. Using an unsuitable conversion may lead to seriously biased MASEM estimates. Moreover, if the group distribution in the sample does not reflect the prevalence in the population, it is necessary to adjust the Pearson product–moment correlation between the continuous variables in the model. To assist researchers with their conversions and adjustments, we extended our freely available web application ESCACO (https://hdejonge.shinyapps.io/ESCACO/), which supports a variety of meta-analytic objectives when the wish is to incorporate a dichotomous or dichotomized variable in their (MASEM) analyses.

## Appendix A

### Population values depending on the prevalence

This appendix explains the values of the population coefficients that we used in our simulation study. We assumed an infinite (super)population existing of two infinite groups (e.g., having a specific disease or not). However, the one group being a certain factor larger than the other, depending on the prevalence of level 1 of the dichotomous variable in the population (e.g., prevalence of a specific disease). For example, if we have a prevalence of 2%, then we assumed parameter $\pi$ (see main text) to be equal to .02. Furthermore, like in RCTs, we assumed the groups to be equal in other aspects, including equal standard deviations. Hence, variable X (status; levels 1 and 0) was assumed to establish only group mean differences (in both M and Y). The group means on variable M were assumed to be μ_M,1_ = 0.40 and μ_M,0_ = – 0.40, and the within-group variance ${\upsigma }_{\mathrm{M},1}^{2}={\upsigma }_{\mathrm{M},0}^{2}={\upsigma }_{\mathrm{M}}^{2}$ = 1, which implies a standardized group mean difference in M of ${\delta }_{\mathrm{M}}=\frac{{\upmu }_{\mathrm{M},1}-{\upmu }_{\mathrm{M},0}}{{\upsigma }_{\mathrm{M}}}=\frac{0.40--0.40}{1}=0.80$, denoting a large effect according to Cohen (1988)’s criteria. The total mean of variable M becomes $\mu_\mathrm{M,T} = \pi \cdot \mu_\mathrm{M,1} + (1 - \pi) \cdot \mu_\mathrm{M,0} = .02 \cdot .40+ .98 \cdot -.40=- 0.384$. Similarly, we assumed μ_Y,1_ = 0.25, μ_Y,0_ = – 0.25 and variance ${\upsigma }_{\mathrm{Y},1}^{2}={\upsigma }_{\mathrm{Y},0}^{2}={\upsigma }_{\mathrm{Y}}^{2}=1$ such that μ_Y,T_ = -0.240 and ${\delta }_{\mathrm{Y}}=\frac{0.25--0.25}{1}=0.50$, denoting a medium effect (Cohen, 1988). Lastly, we assumed within-group correlation between M and Y of $\rho =.40$ (in both groups). Given variable X only induces a shift in mean, this implies total variances (i.e., the variance when groups are combined) of:

$$\begin{array}{c}{\sigma }_{\mathrm{M},\mathrm{T}}^{2}=\pi \left({\sigma }_{\mathrm{M},1}^{2}+{\left({\mu }_{\mathrm{M},1}-{\mu }_{\mathrm{M},\mathrm{T}}\right)}^{2}\right)+\left(1-\pi \right)\left({\sigma }_{\mathrm{M},0}^{2}+{\left({\mu }_{\mathrm{M},0}-{\mu }_{\mathrm{M},\mathrm{T}}\right)}^{2}\right)\\ =.02\left(1+{\left(0.40--0.384\right)}^{2}\right)+\left(1-.02\right)\left(1+{\left(-0.40--0.384\right)}^{2}\right)=1.012544\end{array}$$

and

$$\begin{array}{c}{\sigma }_{\mathrm{Y},\mathrm{T}}^{2}=\pi \left({\sigma }_{\mathrm{Y},1}^{2}+{\left({\mu }_{\mathrm{Y},1}-{\mu }_{\mathrm{Y},\mathrm{T}}\right)}^{2}\right)+\left(1-\pi \right)\left({\sigma }_{\mathrm{Y},0}^{2}+{\left({\mu }_{\mathrm{Y},0}-{\mu }_{\mathrm{Y},\mathrm{T}}\right)}^{2}\right)\\ =.02\left(1+{\left(0.25--0.240\right)}^{2}\right)+\left(1-.02\right)\left(1+{\left(-0.25--0.240\right)}^{2}\right)=1.0049\end{array}$$

As a result, the total correlation between M and Y, ${\rho }_{\mathrm{YM}}$, becomes:

$$\begin{array}{c}{\rho }_{\mathrm{YM}}=\frac{\pi \left(\rho +\left({\mu }_{\mathrm{M},1}-{\mu }_{\mathrm{M},T}\right)\left({\mu }_{\mathrm{Y},1}-{\mu }_{\mathrm{Y},T}\right)\right)+\left(1-\pi \right)\left(\rho +\left({\mu }_{\mathrm{M},0}-{\mu }_{\mathrm{M},T}\right)\left({\mu }_{\mathrm{Y},0}-{\mu }_{\mathrm{Y},T}\right)\right)}{{\sigma }_{\mathrm{Y},T}{\sigma }_{\mathrm{M},T}}\\ =\frac{.02(0.40+\left(0.40--0.384\right)\left(0.25--0.240\right))+\left(1-.02\right)\left(0.40+(-0.40--0.384\right)\left(-0.25--0.240\right))}{\sqrt{1.012544}\sqrt{1.0049}}=.4043165.\end{array}$$

Given the parameter values $\pi =.02$, ${\delta }_{\mathrm{M}}=0.80,$ and ${\delta }_{\mathrm{Y}}=0.50$, the point-biserial correlations in the population become:

$${\rho }_{\mathrm{pb},\mathrm{MX}}=\frac{{\delta }_{\mathrm{M}}}{\sqrt{{\delta }_{\mathrm{M}}^{2}+ \frac{1}{\pi (1-\pi )}}}=\frac{0.80}{\sqrt{{0.80}^{2}+ \frac{1}{.02(1-.02)}}}=.1113041.$$

and

$${\rho }_{\mathrm{pb},\mathrm{YX}}=\frac{{\delta }_{\mathrm{Y}}}{\sqrt{{\delta }_{\mathrm{Y}}^{2}+ \frac{1}{\pi (1-\pi )}}}=\frac{0.5}{\sqrt{{0.5}^{2}+ \frac{1}{.02(1-.02)}}}=.06982913.$$

Given the path model (see Fig. 1 in the main document) and the correlations ${\rho }_{\mathrm{pb},\mathrm{MX}}\;=\;.1113041$, ${\rho }_{\mathrm{pb},\mathrm{YX}}\;=\;.06982913$, and ${\rho }_{\mathrm{YM}}\;=\;.4043165$ the path coefficients equal:

$$\begin{array}{c}{\beta }_{\mathrm{MX}}= {\rho }_{pb,\mathrm{MX}}=.1113041,\\ {\beta }_{\mathrm{YX}}= \frac{{\rho }_{\mathrm{pb},\mathrm{MX}}{\rho }_{\mathrm{YM}}-{\rho }_{\mathrm{pb},\mathrm{YX}}}{{\rho }_{\mathrm{pb},\mathrm{MX}}^{2}-1}=\frac{ .1113041 \cdot .4043165 - .06982913.}{{ .1113041}^{2} - 1}=.02513848,\text{ and}\\ {\beta }_{\mathrm{YM}}= \frac{{\rho }_{\mathrm{pb},\mathrm{MX}}{\rho }_{\mathrm{pb},\mathrm{YX}}-{\rho }_{\mathrm{YM}}}{{\rho }_{\mathrm{pb},\mathrm{MX}}^{2}-1}=\frac{.1113041 \cdot .06982913-.4043165}{{.1113041}^{2}-1}=.4015185.\end{array}$$

The indirect effect of X on Y becomes: ${\beta }_{\mathrm{MX}}{\cdot \beta }_{\mathrm{YM}}= .1113041\cdot.4015185 = .04469064$.

For the other prevalences in this study (i.e., .10 and .50), one can fill in these values for $\pi$ instead of .02. This will lead to the population values of the correlations, path coefficients, and indirect effect as provided in Table 9 in this appendix.

**Table 9:** Population values of the correlations, path coefficients, and indirect effect belonging to the different prevalences in this study

	ρ_pb,MX_	ρ_pb,YX_	ρ_YM_	${\upbeta }_{\mathrm{MX}}$	${\upbeta }_{\mathrm{YX}}$	${\upbeta }_{\mathrm{YM}}$	Indirect effect $({\upbeta }_{\mathrm{MX}}\cdot {\upbeta }_{\mathrm{YM}})$
π = .02	.111	.070	.404	.111	.025	.402	.045
π = .10	.233	.148	.419	.233	.053	.407	.095
π = .50	.371	.243	.450	.371	.087	.418	.155

## Data Availability

All R scripts needed to reproduce the simulations, simulated data, and Shiny Application can be found on the Open Science Framework (OSF; https://osf.io/mkv65/).

## Footnotes

1 In this paper, the term population base rate can be considered synonymous with prevalence.

2 Note that these sources may differ in the notation of this formula.

3 There has been some criticism of the use of Cohen's rules of thumb (e.g., Funder & Ozer, 2019). However, the rules of thumb are still widely used in practice.

4 One only need to provide correlation matrices and sample sizes to the R package metaSEM. There is no need to provide, for instance, the sampling variances or standard errors of the correlations, which are required in the R package metafor. The R package metaSEM models the variance and covariance matrix of the correlations in the background.

5 We note that because the partial mediation model is a saturated model, the model-implied correlations are equal to the pooled correlations. In practice, when using the osmasem() function in the metaSEM R package, the pooled correlations are not part of the output. If one wishes to obtain the pooled correlations in other (non-saturated) models, this can be done using the first stage of two-stage structural equation modeling, which is implemented in the tssem1() function in metaSEM.

## Code availability

All R scripts needed to reproduce the simulations, simulated data, and Shiny Application can be found on the Open Science Framework (OSF; https://osf.io/mkv65/).
